# α-synuclein and p53 functional interplay in physiopathological contexts

**DOI:** 10.18632/oncotarget.14385

**Published:** 2016-12-30

**Authors:** Cristine Alves da Costa, Eric Duplan, Frédéric Checler

**Affiliations:** Université Côte d’Azur, INSERM, CNRS, IPMC, team labeled “Laboratory of Excellence (LABEX) Distalz”, Sophia-Antipolis, Valbonne, France

**Keywords:** Parkinson’s disease, α-synuclein, p53, apoptosis, transcription, Neuroscience

Parkinson’s disease (PD) is the most frequent age-related movement disorder due to the loss of dopaminergic neurons. It is characterized at the histological level by the presence of intracellular lesions named Lewy bodies and cell death stigmata. The main constituent of Lewy bodies is α-synuclein (α-syn), a phosphoprotein that is strongly linked to the etiology of both genetic and sporadic PD. α-syn is a protein contributing to dopaminergic neuronal health and numerous evidences indicate that, in physiological conditions α-syn is neuroprotective while, in PD-related pathological conditions, it becomes aggregated and thereby, neurotoxic[1, 2].

More than ten years ago, we were first to show that wild-type α-syn displayed antiapoptotic properties (OMIM entry 163890) and that this function was abolished by its aggregation [3, 4]. We showed that α-syn overexpression in telencephalon mouse neurons led to decreased cell death via the down-regulation of the tumor suppressor p53 (a key transcription factor involved in apoptosis regulation in neurodegenerative disorders [5, 6]) at both protein and transcriptional levels and that this phenotype was abolished by the pro-oxidant neurotoxin and PD-inducer, 6-hydroxydopamine (6OHDA) [4]. Importantly, we have demonstrated that 6OHDA triggers α-syn loss of function by modifying its biochemical properties and catabolic fate [7]. This was explained by our two-points demonstration that: 1) 6OHDA pro-oxidant properties promote *in vitro* α-syn aggregation; 2) 6OHDA-mediated oxidation abolishes *in vitro* and cellular proteasomal activities and thus, proteasome-mediated α-syn degradation. Therefore, the accumulation of α-syn by 6OHDA-mediated proteasomal inhibition leads to α-syn aggregation and dysfunction. These seminal observations gave an anatomical support to the paradoxical observation that α-syn aggregation occurs selectively in dopaminergic brain regions (i.e regions where 6OHDA can be produced) while α-syn is widely spread over whole brain. This led us to propose that PD, at least in sporadic cases, is much more related to a cell-specific susceptibility to dopaminergic toxins rather than linked to the presence of a subset of pathological protein triggers.

We have recently demonstrated that the functional interplay by which α-syn controls p53 is not unidirectional and is controlled by a feedback loop. Thus, endogenous and overexpressed p53 activate α-syn transcription *ex-vivo* [8] and both pharmacological and genetic manipulations of p53 increased α-syn protein, promoter activity and mRNA levels. We demonstrated that α-syn is a genuine p53 target by combined and complementary approaches. First, bioinformatics analysis of the mouse α-syn promoter led us to identify a p53 responsive element, the deletion of which fully abolished p53-mediated α-syn promoter transactivation. Second, gel shift analysis of bimolecular reactions between recombinant p53 and biotin-labeled DNA probes encompassing the p53 responsive element on mouse α-syn promoter unraveled a physical interaction between p53 and α-syn without the participation of any additional co-factor. Third, chromatin immunoprecipitation assay (ChiP) demonstrated that this interaction also occurs in physiological conditions. Importantly, we showed that this regulation takes place *in vivo* since brain samples from p53 knockout mice display increased α-syn protein and mRNA levels compared to age-matched controls.

**Figure 1:**
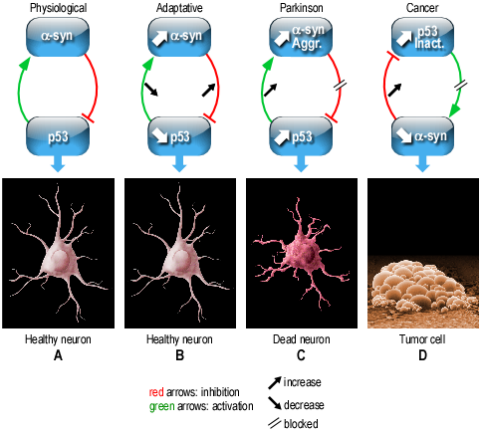
Interplay between α-synuclein and p53 in normal and pathological contexts

Overall, our data indicate a regulatory loop between α-syn and p53 that can have distinct consequences in physiological and pathological contexts. These multiple p53-α-syn interplays are resumed in Figure 1. Thus, in physiological conditions, the functional interplay between p53 and α-syn drives the homeostasis of the two protein partners (Figure 1A). This physiological control has to adapt to mild cellular challenge in order to prevent systematic cell wounding. Thus, upon mild stress (Figure 1B), any up/or down modulation of either α-syn or p53 is thwarted by an opposite variation of its partner aimed at restoring physiological levels of initially altered protein. In the example shown in B, an increase in α-syn lowers p53 that in turn, reduces α-syn levels, thereby counteracting initial α-syn enhancement.

In chronic pathological contexts (Figure 1C, 1D), the functional interplay between α-syn and p53 is perturbed and cannot compensate for cellular alterations. Thus, in a PD pathological context, α-syn loss of function triggered for example, by its aggregation (Aggr), will enhance p53 expression that will increase α-syn levels, feed its aggregation and inactivation (Inact.) and consequently, increase cell death. A defective interplay between these proteins can also be envisioned in additional pathological chronic contexts. For example in cancer, p53 is frequently inactivated by mutations. Thus p53 loss of function should lead to α-syn reduction that in turn, should feed accumulation of dysfunctional (mutated or biochemically modified) p53. Overall, this physiological functional interplay between α-syn and p53 that can convert into a vicious cycle, could well, at least partly, underlie mechanistic dysfunctions taking place in both neurodegenerative diseases such as PD and cancer.
